# Pediatric Cullen gangrene: Case report of postoperative pyoderma gangrenosum in a preterm infant with a complex gastrointestinal malformation

**DOI:** 10.1016/j.ijscr.2019.12.037

**Published:** 2020-01-09

**Authors:** Sonja Diez, Julia Syed, Hanna Müller, Manuel Besendoerfer, Vera Schellerer

**Affiliations:** a Pediatric Surgery, Surgery Department, University Hospital Erlangen, Loschgestrasse 15, Erlangen, Germany; b Children’s Hospital Erlangen, Neonatology and Pediatric Intensive Care Unit, University Hospital Erlangen, Loschgestrasse 15, Erlangen, Germany

**Keywords:** PG, pyoderma gangrenosum, PPG, postoperative pyoderma gangrenosum, NICU, neonatal intensive care unit, VRE, vancomycin resistant enterococcus, Cullen gangrene, Pyoderma gangrenosum, Gastroschisis, Pediatric surgery

## Abstract

**Introduction:**

Pyoderma gangrenosum (PG) is a rare condition in infants, children and adolescents. The postoperative variant of this rapidly progressive ulceration is known as Cullen gangrene. Only a few case reports of pediatric Cullen gangrene have been published.

**Presentation of case:**

We present a male infant (gestational age 31 + 6 weeks) with gastroschisis and intrauterine volvulus. After multiple operations due to his complex gastrointestinal malformation, the patient showed classical symptoms of postoperative PG (PPG) 4 weeks after birth. Diagnosis could be made early in an interdisciplinary approach, differentiating intraabdominal infection (vancomycin resistant enterococcus (VRE), candida albicans) from epifascial occurrence. Treatment with high-dose steroids and immunoglobulins was initiated immediately and led to rapid recovery.

**Discussion:**

The unusual combination of a complex gastrointestinal malformation and an intraabdominal infection with VRE and candida albicans made the diagnosis difficult. However, early diagnosis is crucial in the treatment of Cullen gangrene.

**Conclusion:**

This is the first case report of Cullen gangrene presenting in an infant (at 4 weeks of age).

## Highlights

• Postoperative pyoderma gangrenosum is a rare condition in children.
• Diagnosis is based on exclusion of other diseases.
• An intraabdominal infection or other diseases can occur simultaneously.
• Early diagnosis and treatment are evident to optimal patient’s care.

## 1 Introduction

Pyoderma gangrenosum (PG) is an uncommon neutrophilic dermatosis with an average incidence of 3–10 per million patients per year. This chronic dermatosis is a rare condition in the pediatric population with only 4–5 percent of PG occurring in children, whereas adults are most commonly affected in the third to sixth decades of life [1,2]. In 50–78% of patients, there is an association with immunological diseases such as rheumatoid arthritis, chronic inflammatory bowel disease [3], or a paraneoplastic background [4].

The Cullen gangrene is considered as its postoperative pathergic version (PPG, postoperative pyoderma gangrenosum), occurring solely at the surgical sites [5]. Association to systemic diseases could not be seen in this postoperative subtype of PG [6,7], and so far, risk factors for its development have not been identified.

In a systematic review, Zuo et al. presented only three children out of 220 analyzed patients with PPG [7]. We present the management of a preterm infant at the age of 4 weeks with a fulminant clinical course of PPG at a level one perinatal center, which could be managed because of an early diagnosis. The work has been reported in line with the SCARE criteria [8].

## 2 Presentation of case

**Fig. 1:**
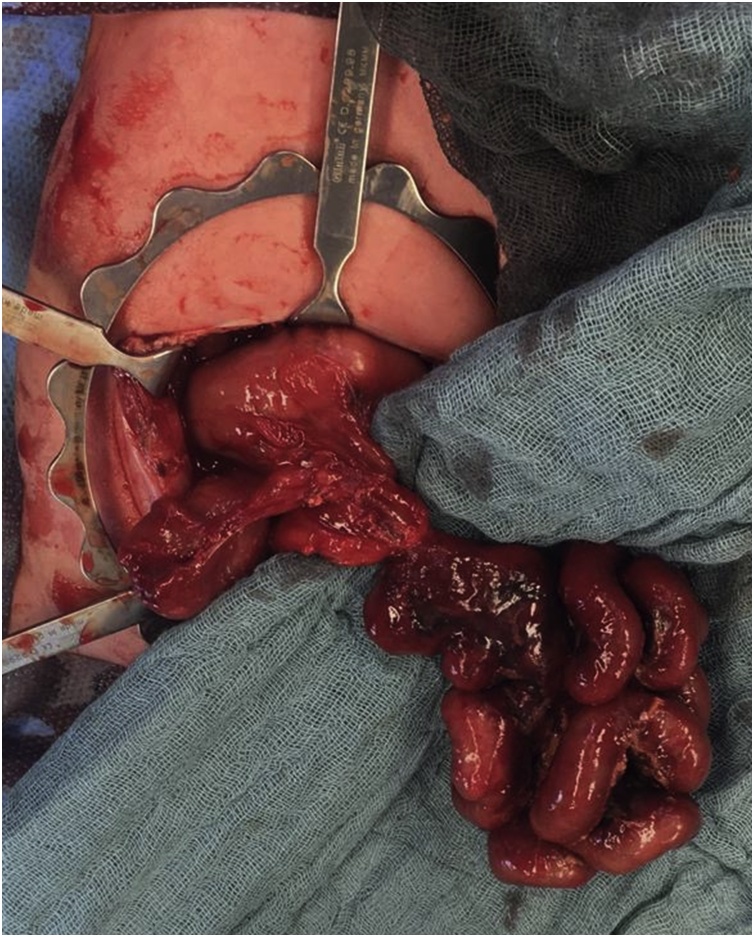
Intraoperative findings of the second surgery, presenting a complex gastrointestinal malformation, including an intrauterine volvulus and accordingly twisted mesenteric vessels in combination with a duodenal atresia and atresia of the ascending colon. Ischemic discoloration was already notable (surgery at an absolute age of 5 days).

A newborn male infant of 31 + 6 weeks of gestational age and a birth weight of 2300 g was introduced to our pediatric surgery department at his fourth day of life. After a natural birth, the patient presented with a large abdominal wall defect, undetected in prenatal ultrasonography. After initial treatment of gastroschisis with silo placement in an external clinic, the patient was transferred to our center for further therapy. Abdominal closure as a final repair of gastroschisis defect was scheduled at the fifth day of life. Exploration of intraabdominal organs confirmed a complex gastrointestinal malformation, including duodenal atresia and atresia of the ascending colon. It can be assumed that the intrauterine volvulus and the resultant twisted mesenteric vessels have led to a long-distance loss of the small intestine (see intraoperative findings in Fig. 1). Intestinal integrity was restored by duodeno-jejunostomy and a colostomy, with secondary closure of the abdominal wall.

The patient was transferred to the NICU. Ventilation and sedation could be terminated after 5 days and oral nutrition was started carefully and was well tolerated.

After three weeks at an absolute age of 24 days, the patient presented with a rapidly progressive, septic deterioration. An acute abdomen and clinical signs of an abdominal compartment syndrome developed, as well as respiratory and hemodynamic failure.

CRP levels showed increased values of 106 mg/l. At an emergency re-laparotomy, ischemic necrosis of small bowel and caecum with a subhepatic abscess were detected and managed by subtotal removal of intestine and drainage of pus. The patient was managed with total parenteral nutrition.

Postoperative management at NICU remained challenging, as sepsis caused remaining respiratory failure, capillary leak and hemodynamic instability. Intraoperative microbiological swabs detected an intraabdominal infection with vancomycin resistant enterococcus (VRE) and candida albicans. Antibiotic treatment was therefore extended to linezolid, meropenem and fluconazole on the third postoperative day leading to clinical improvement of the patient.

**Fig. 2:**
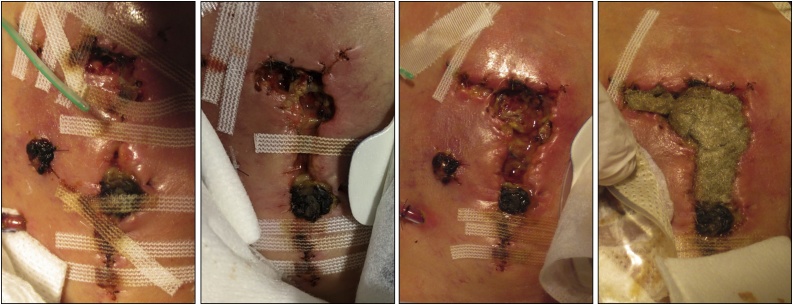
Clinical presentation of the ulcerous development of the Cullen gangrene in the presented infant. First picture was taken after one day of abdominal erythema. Every further picture shows development after 12 hours, indicating enlargement of the lesion to a size measuring 5 × 3 cm of maximal diameters.

On the fourth postoperative day, painful abdominal erythema, edema and exsudation were noted. There was extensive leucocythemia and CRP levels increased tremendously to 290 mg/l. Within 48 h, the sutures dissolved and a major wound dehiscence occurred (see Fig. 2). The subcutaneous tissue was noted to be devitalized and largely dissolved, although the fascia did not seem to be affected. No fever occurred.

**Fig. 3:**
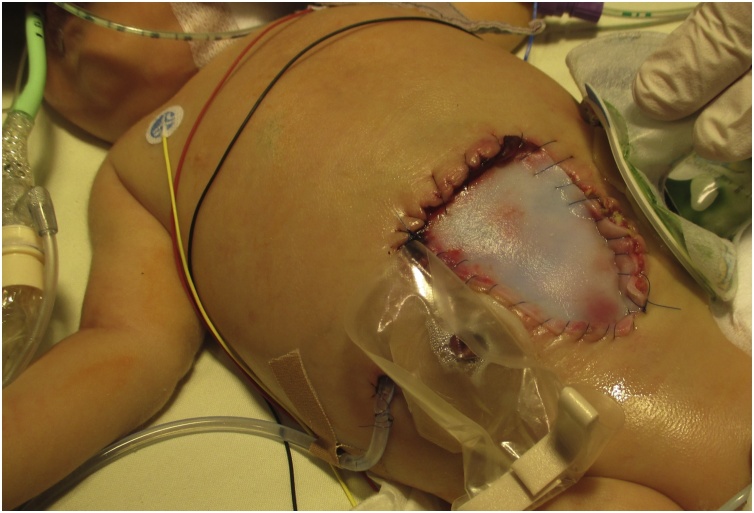
After surgery for biopsies and careful exploration of wound defect, secondary closure via biological patch was performed. The cutaneous edges were not excised, but tension-free attached to the patch.

**Fig. 4:**
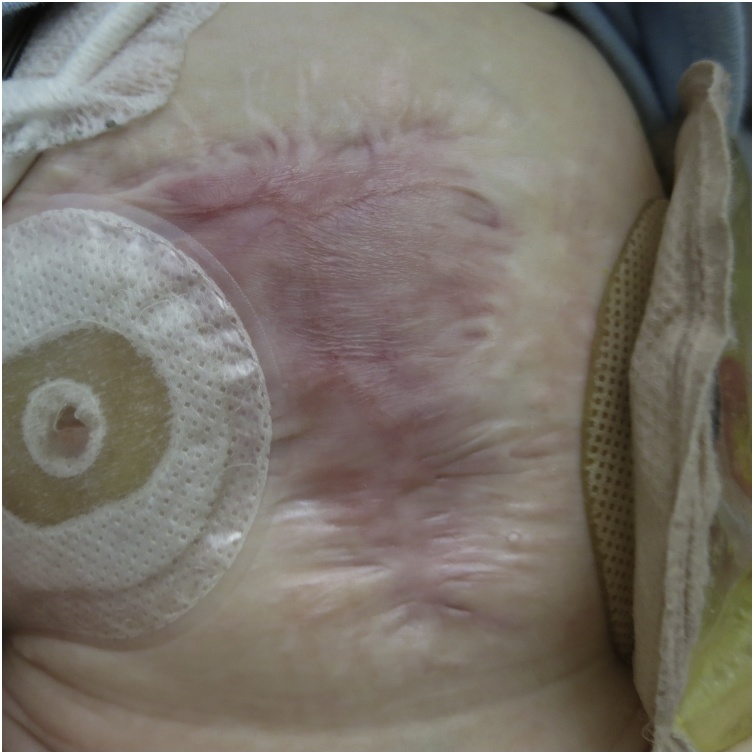
The infant’s abdominal wall at the age of 7 months after secondary closure of abdominal wall. Therapy with low-dose prednisolone was continued to this time.

In an interdisciplinary cooperation of pediatric surgeons, neonatologists and dermatologists, the diagnosis of Cullen gangrene was suspected and treatment was thus determined. In the following surgery at fourth day of clinical symptoms, manipulation and debridement of tissue was kept at a minimum to avoid further pathergy effects. The abdominal closure was therefore completed with a biological patch (see Fig. 3). The skin biopsies taken intraoperatively confirmed the suspected neutrophilic dermatosis. Treatment with systemic high-dose corticosteroids (4 mg/kg/d) and intravenous immunoglobulins (1 g/kg/d for 6 days) was initiated immediately, maintaining a postoperative stable cutaneous condition and a prompt proper wound healing within the following weeks. Prednisolone was slowly tapered to a dose of 0.1 mg/kg/d over 10 weeks. A trial discontinuation of medication after 12 weeks led to an immediate relapse, which responded to resumption of therapy. Prophylactic steroid therapy was given for 8 months (0.5 mg/kg/d). Further local relapse of disease did not occur. Final picture of the infant’s abdominal wall is shown in Fig. 4 at an age of 7 months.

## 3 Discussion

We present the first published case of PPG in an infant of 4 weeks of age. Further reports on the characteristics and the optimal treatment in children and adolescents have been published sporadically over the past 20 years [[9], [10], [11]], and the optimal therapy in this age group is clearly a challenge for the attending surgeons and neonatologists. However, pediatric PPG has been treated successfully according to the adult regimen proposed in several reviews so far [6,7,12].

Case reports in children are presenting an early occurrence within 4 postsurgical days [9]. Description of clinical characteristics is outlining the initial erythema, severe pain and wound dehiscence of PPG. Cutaneous papulo-pustules are rapidly evolving into a superficial, necrotic wound with undermined, violaceous edges. Accordingly, our patient showed no involvement of the abdominal fascia. A strong systemic reaction, including fever and elevated inflammatory blood values (CRP, leukocytosis with a high neutrophil count), can be seen within its occurrence [7], which we can confirm.

A multifactorial pathogenesis has been broadly discussed. The up-regulation of polymorphonuclear neutrophils via cytokines (interleukin-8, -12, -23) leads to an autoimmune regulated neutrophilic dysfunction and impaired phagocytosis [13]. Additional genetic variants are proposed with an influence on pathogenesis, yet this remains controversial [14].

The surgical trauma is assumed to be the initiating factor of pathergy in PPG. Pathergy usually results from an iatrogenic trauma, introducing an inflammatory process via cytokine release and finally leading to a local deterioration. This phenomenon is more common in childhood than in adults [10].

The cutaneous inflammatory behavior is often the cause for misdiagnosis of local wound infections. Additionally, the necrotic lesions can strongly resemble gangrene of necrotizing fasciitis and various other diseases with rapidly evolving wound deterioration and laboratory findings as in septicemia [12]. Differential diagnosis is thus essential to an optimal treatment, as antibiotic treatment proved no effect on this entity and surgical wound debridement as treatment for a presumptive necrotizing fasciitis may lead to rapid ulcer enlargement due to pathergy with severe consequences [11]. Histopathological examination is thus important to exclude other diseases. However, as there is a non-specific inflammatory infiltrate and a surrounding neutrophilic and lymphocytic component [12], the formal diagnosis of PPG remains one of exclusion, centered on the clinical appearance and response to immunomodulatory therapy [15].

Diagnosis in the presented case could be made according to the diagnostic criteria of Su et al. [15], although differential diagnosis was challenging due to the parallel course of a septic intraabdominal infection with VRE and candida albicans and the epifascial PPG. With VRE being essentially involved in wound infections, discrimination of these two processes had to be made carefully. But the ulcerative abdominal lesion developed and aggravated under high dose antibiotic treatment, readjusted according to the tested antibiogram. A postoperative wound infection could therefore be excluded. In this case, an early diagnosis was able to impede severe consequences for the infant.

Therapeutic options for PPG and PG are similar and based on principals of immunosuppression. Systemic corticosteroids (prednisolone, 1–2 mg/kg/d) and cyclosporine (2–5 mg/kg/d) alone or in combination are proposed as first-line therapies in literature [7].

Improvement may be seen within the first 24 h. Furthermore, different drugs, such as immunoglobulins, dapsone, sulphasalazine, mercaptopurine, azathioprine, mycophenolate mofetil, cyclophosphamide, infliximab and tacrolimus have been recently considered as additional or substitutional options [16]. The use of these drugs certainly is limited due to admission regulation in neonatology. Surgical treatment plays a subordinate role in clinical management and remains to be discussed controversially. However, in combination with immunomodulators after an initial stabilization, skin grafting, flap coverage or a cautious debridement may add positive effects, which we can confirm with this clinical presentation.

Relapse rates are high in PG/PPG. In order to prevent recurrence of disease, a prophylactic steroid therapy is recommended, which should be tapered gradually over 6 months [7]. We decided to enlarge therapy over 8 months, because of complex clinical course and first signs of relapse in a trail discontinuation of medication.

## 4 Conclusion

This is the first case report of an infant of 4 weeks of age with PPG which followed a severe, complex clinical course. No such combination of Cullen gangrene, with either a gastrointestinal malformation or an intraabdominal infection, has been published to date. Early diagnosis in combination with high-dose therapy and a careful surgical approach can be considered crucial to optimal treatment. Cullen gangrene in infants, children and adolescents remains a rare condition. However, pediatric surgeons should bear this condition in mind, as false therapeutic trials may have serious adverse effects on the clinical course in affected patients and on their quality of life.

## Funding

This research did not receive any specific grant from funding agencies in the public, commercial, or not-for-profit sectors.

## Ethical approval

The study is exempt from ethnical approval in our institution.

## Consent

Written informed consent was obtained from the patient’s parents for publication of this case report and accompanying images. This report does not contain any personal information that could lead to the identification of the patient.

## Author contribution

Sonja Diez: corresponding author, collection of data, conception and revision of the manuscript.

Julia Syed: the surgeon who run the operation, acquisition of data, review of PubMed Library and review of the manuscript.

Hanna Müller: review of the PubMed Library, revision of the manuscript.

Manuel Besendörfer: garantor, review of the manuscript.

Vera Schellerer: the surgeon run the operation, acquisition of data, and final approval of the version to be submitted, garantor.

## Registration of research studies

Our manuscript is a case report not a research.

## Guarantor

Dr. med. Vera Schellerer.

Dr. med. Manuel Besendörfer.

## Provenance and peer review

Editorially reviewed, not externally peer-reviewed.

## Declaration of Competing Interest

Non to declare.
